# Bis(μ-quinoline-2-carboxyl­ato)-κ^3^ *N*,*O*:*O*;κ^3^ *O*:*N*,*O*-bis­[(acetato-κ^2^ *O*,*O*′)(methanol-κ*O*)lead(II)]

**DOI:** 10.1107/S1600536810027777

**Published:** 2010-07-17

**Authors:** Gholamhossein Mohammadnezhad, Ali Reza Ghanbarpour, Mostafa M. Amini, Seik Weng Ng

**Affiliations:** a Department of Chemistry, General Campus, Shahid Beheshti University, Tehran 1983963113, Iran; b Department of Chemistry, University of Malaya, 50603 Kuala Lumpur, Malaysia

## Abstract

The dinuclear title compound, [Pb_2_(C_10_H_6_NO_2_)_2_(CH_3_COO)_2_(CH_3_OH)_2_], lies across an inversion center. The methanol-coordinated Pb^II^ atom is chelated by the acetate anion as well as by the quinoline-2-carboxyl­ate anion. One O atom of the quinoline-2-carboxyl­ate anion bridges two symmetry-related Pb^II^ atoms, forming the dinuclear compound. Aside from the six atoms connected to the Pb^II^ atom by regular coordination bonds, the structure features a long Pb⋯O inter­action [3.145 (3) Å] that gives rise to a distorted Ψ-square-anti­prismatic geometry at the metal center. The H atom of the methanol is hydrogen bonded to an O atom of the acetate.

## Related literature

For a related structure, see: Mohammadnezhad *et al.* (2010 ▶).
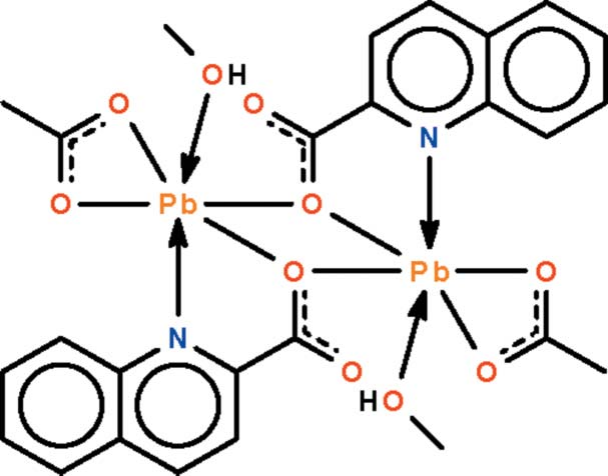


## Experimental

### 

#### Crystal data

[Pb_2_(C_10_H_6_NO_2_)_2_(C_2_H_3_O_2_)_2_(CH_4_O)_2_]
*M* *_r_* = 940.87
Monoclinic, 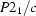

*a* = 7.3197 (3) Å
*b* = 8.3065 (4) Å
*c* = 23.3247 (10) Å
β = 90.397 (1)°
*V* = 1418.13 (11) Å^3^
*Z* = 2
Mo *K*α radiation
μ = 11.91 mm^−1^
*T* = 100 K
0.25 × 0.15 × 0.10 mm

#### Data collection

Bruker SMART APEX diffractometer
Absorption correction: multi-scan (*SADABS*; Sheldrick, 1996 ▶) *T*_min_ = 0.155, *T*_max_ = 0.382
13298 measured reflections
3267 independent reflections
3055 reflections with *I* > 2σ(*I*)
*R*_int_ = 0.025

#### Refinement

*R*[*F* ^2^ > 2σ(*F* ^2^)] = 0.019
*wR*(*F* ^2^) = 0.049
*S* = 1.06
3267 reflections
186 parameters
1 restraint
H atoms treated by a mixture of independent and constrained refinement
Δρ_max_ = 1.65 e Å^−3^
Δρ_min_ = −1.43 e Å^−3^

### 

Data collection: *APEX2* (Bruker, 2009 ▶); cell refinement: *SAINT* (Bruker, 2009 ▶); data reduction: *SAINT*; program(s) used to solve structure: *SHELXS97* (Sheldrick, 2008 ▶); program(s) used to refine structure: *SHELXL97* (Sheldrick, 2008 ▶); molecular graphics: *X-SEED* (Barbour, 2001 ▶); software used to prepare material for publication: *publCIF* (Westrip, 2010 ▶).

## Supplementary Material

Crystal structure: contains datablocks global, I. DOI: 10.1107/S1600536810027777/xu2798sup1.cif

Structure factors: contains datablocks I. DOI: 10.1107/S1600536810027777/xu2798Isup2.hkl

Additional supplementary materials:  crystallographic information; 3D view; checkCIF report

## Figures and Tables

**Table 1:** Selected bond lengths (Å)

Pb1—O1	2.377 (3)
Pb1—O1^i^	2.490 (2)
Pb1—O2^ii^	3.145 (3)
Pb1—O3	2.382 (3)
Pb1—O4	2.761 (3)
Pb1—O5	2.696 (3)
Pb1—N1	2.643 (3)

Symmetry codes: (i) 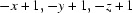; (ii) 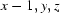.

**Table 2:** Hydrogen-bond geometry (Å, °)

*D*—H⋯*A*	*D*—H	H⋯*A*	*D*⋯*A*	*D*—H⋯*A*
O5—H5⋯O3^i^	0.84 (4)	1.89 (4)	2.685 (4)	158 (5)

Symmetry code: (i) 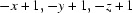.

**Fig. 1.**
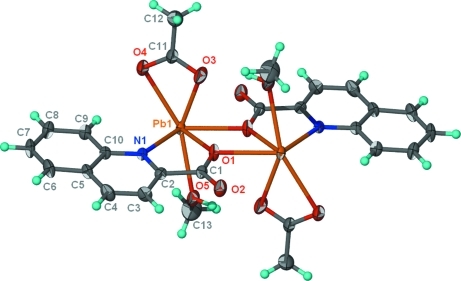
Thermal ellipsoid plot (Barbour, 2001) of [Pb(CH4O)2(C2H3O2)2(C10H6NO2)2]2 at the 70% probability level. Hydrogen atoms are drawn as spheres of arbitrary radius. The dinuclear molecule lies on a center-of-inversion.

**Fig. 2.**
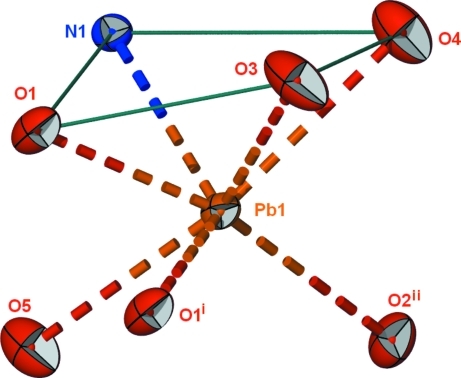
Ψ-Square antiprismatic geometry of lead. Symmetry codes are given in Table 1.

## supplementary crystallographic information

### Comment

The lead(II) atom in Pb(C_10_H_6_NO_2_)_2_ is *N*,*O*-chelated by the carboxylate anions in a Ψ-trigonal bipyramidal environment; four atoms are connected to the lead atom by regular coordination bonds (Mohammadnezhad *et al.*, 2010). This compound was synthesized by the reaction of lead acetate and quinoline-2-carboxylic acid in the presence of potassium nitrite. With potassium nitrate in place of potassium nitrite, the synthesized yielded instead dinuclear [Pb(CH_4_O)_2_(C_2_H_3_O_2_)_2_(C_10_H_6_NO_2_)_2_]_2_ (Scheme I, Fig. 1) in which only one of the acetate groups is replaced by the quinoline-2-carboxylate group. The methanol-coordinated lead(II) atom is chelated by the acetate anion as well as by the quinoline-2-carboxylate anion; the oxygen atom involved in chelation is also datively coordinated to the symmetry-related lead atom. When a long Pb···O2^ii^ (*ii* = *x* - 1, *y*, *z*) interaction of 3.145 (3) Å is considered to be a bond, the geometry is a Ψ-antiprism (Fig. 2).

### Experimental

Lead(II) acetate (1 mmol, 0.38 g), quinoline-2-carboxylic acid (1 mmol, 0.17 g) and potassium nitrate (1 mmol, 0.10 g) were loaded into one arm of a convection tube and both of the arms were filled slowly by methanol. The chemical-bearing arm was immersed in an oil bath kept at 333 K. Crystals were formed on the inside surface of the arm kept at ambient temperature after a weekfew days.

### Refinement

Hydrogen atoms were placed in calculated positions (C–H 0.95–0.98 Å) and included in the refinement in the riding model approximation, with *U*(H) set to 1.2–1.5*U*_eq_(C).

The hydroxy H-atom was located in a difference Fourier map, and was refined with a distance restraint of O–H 0.84±0.01 Å; its temperature factor was refined.

The final difference Fourier map had a peak/hole in the vicinity of Pb1.

### Crystal data

**Table:**

[Pb_2_(C_10_H_6_NO_2_)_2_(C_2_H_3_O_2_)_2_(CH_4_O)_2_]	*F*(000) = 880
*M**_r_* = 940.87	*D*_x_ = 2.203 Mg m^−^^3^
Monoclinic, *P*2_1_/*c*	Mo *K*α radiation, λ = 0.71073 Å
Hall symbol: -P 2ybc	Cell parameters from 8885 reflections
*a* = 7.3197 (3) Å	θ = 2.6–28.3°
*b* = 8.3065 (4) Å	µ = 11.91 mm^−^^1^
*c* = 23.3247 (10) Å	*T* = 100 K
β = 90.397 (1)°	Block, colorless
*V* = 1418.13 (11) Å^3^	0.25 × 0.15 × 0.10 mm
*Z* = 2	

### Data collection

**Table:**

Bruker SMART APEX diffractometer	3267 independent reflections
Radiation source: fine-focus sealed tube	3055 reflections with *I* > 2σ(*I*)
graphite	*R*_int_ = 0.025
ω scans	θ_max_ = 27.5°, θ_min_ = 1.8°
Absorption correction: multi-scan (*SADABS*; Sheldrick, 1996)	*h* = −9→9
*T*_min_ = 0.155, *T*_max_ = 0.382	*k* = −10→10
13298 measured reflections	*l* = −30→29

### Refinement

**Table:**

Refinement on *F*^2^	Primary atom site location: structure-invariant direct methods
Least-squares matrix: full	Secondary atom site location: difference Fourier map
*R*[*F*^2^ > 2σ(*F*^2^)] = 0.019	Hydrogen site location: inferred from neighbouring sites
*wR*(*F*^2^) = 0.049	H atoms treated by a mixture of independent and constrained refinement
*S* = 1.06	*w* = 1/[σ^2^(*F*_o_^2^) + (0.0252*P*)^2^ + 2.8416*P*] where *P* = (*F*_o_^2^ + 2*F*_c_^2^)/3
3267 reflections	(Δ/σ)_max_ = 0.001
186 parameters	Δρ_max_ = 1.65 e Å^−^^3^
1 restraint	Δρ_min_ = −1.43 e Å^−^^3^

### Fractional atomic coordinates and isotropic or equivalent isotropic displacement parameters (Å^2^)

**Table:**

	*x*	*y*	*z*	*U*_iso_*/*U*_eq_	
Pb1	0.271223 (16)	0.534286 (15)	0.449130 (5)	0.01353 (5)	
O1	0.5946 (3)	0.5505 (3)	0.45723 (11)	0.0199 (5)	
O2	0.8654 (3)	0.6518 (3)	0.43362 (11)	0.0240 (6)	
O3	0.3648 (4)	0.2628 (4)	0.43453 (12)	0.0313 (7)	
O4	0.1736 (4)	0.3174 (3)	0.36464 (12)	0.0294 (6)	
O5	0.3561 (4)	0.8211 (4)	0.49639 (12)	0.0284 (6)	
H5	0.454 (4)	0.818 (7)	0.515 (2)	0.043*	
N1	0.4435 (4)	0.6512 (3)	0.35907 (12)	0.0142 (5)	
C1	0.7022 (4)	0.6278 (4)	0.42410 (14)	0.0158 (7)	
C2	0.6171 (4)	0.6881 (4)	0.36843 (14)	0.0154 (6)	
C3	0.7256 (5)	0.7731 (5)	0.32939 (16)	0.0212 (7)	
H3	0.8477	0.8020	0.3389	0.025*	
C4	0.6514 (5)	0.8138 (5)	0.27705 (17)	0.0247 (8)	
H4	0.7230	0.8689	0.2495	0.030*	
C5	0.4697 (5)	0.7735 (4)	0.26467 (15)	0.0185 (7)	
C6	0.3834 (5)	0.8108 (5)	0.21153 (16)	0.0252 (8)	
H6	0.4512	0.8628	0.1823	0.030*	
C7	0.2051 (6)	0.7731 (5)	0.20205 (15)	0.0251 (8)	
H7	0.1489	0.8001	0.1665	0.030*	
C8	0.1033 (5)	0.6945 (5)	0.24449 (15)	0.0238 (8)	
H8	−0.0207	0.6672	0.2371	0.029*	
C9	0.1807 (5)	0.6563 (5)	0.29670 (15)	0.0200 (7)	
H9	0.1099	0.6046	0.3253	0.024*	
C10	0.3661 (5)	0.6944 (4)	0.30758 (14)	0.0152 (6)	
C11	0.2681 (5)	0.2208 (5)	0.39130 (15)	0.0213 (7)	
C12	0.2797 (8)	0.0481 (5)	0.3721 (2)	0.0366 (11)	
H12A	0.1787	0.0246	0.3456	0.055*	
H12B	0.2717	−0.0229	0.4056	0.055*	
H12C	0.3962	0.0302	0.3527	0.055*	
C13	0.2189 (7)	0.8980 (7)	0.5289 (2)	0.0478 (13)	
H13A	0.0984	0.8608	0.5160	0.072*	
H13B	0.2276	1.0148	0.5237	0.072*	
H13C	0.2356	0.8717	0.5696	0.072*	

### Atomic displacement parameters (Å^2^)

**Table:**

	*U*^11^	*U*^22^	*U*^33^	*U*^12^	*U*^13^	*U*^23^
Pb1	0.00951 (7)	0.01915 (8)	0.01191 (7)	0.00240 (5)	−0.00103 (4)	0.00037 (5)
O1	0.0138 (12)	0.0310 (15)	0.0148 (12)	0.0017 (10)	−0.0010 (9)	0.0060 (10)
O2	0.0141 (12)	0.0336 (15)	0.0244 (13)	−0.0003 (11)	−0.0035 (10)	0.0041 (11)
O3	0.0334 (16)	0.0291 (15)	0.0313 (15)	0.0141 (13)	−0.0169 (12)	−0.0117 (12)
O4	0.0368 (16)	0.0248 (14)	0.0264 (14)	0.0051 (13)	−0.0145 (12)	−0.0018 (12)
O5	0.0276 (15)	0.0275 (14)	0.0300 (15)	0.0107 (12)	−0.0137 (12)	−0.0051 (12)
N1	0.0136 (13)	0.0167 (14)	0.0123 (13)	0.0012 (11)	−0.0002 (10)	0.0004 (11)
C1	0.0140 (15)	0.0187 (17)	0.0147 (15)	0.0024 (13)	−0.0004 (12)	−0.0010 (13)
C2	0.0140 (15)	0.0158 (16)	0.0164 (16)	0.0025 (13)	0.0001 (12)	−0.0024 (13)
C3	0.0126 (16)	0.0240 (18)	0.0270 (19)	−0.0014 (14)	0.0025 (14)	0.0049 (15)
C4	0.0204 (18)	0.0270 (19)	0.0269 (19)	0.0012 (16)	0.0050 (15)	0.0112 (16)
C5	0.0208 (17)	0.0197 (17)	0.0151 (16)	0.0040 (14)	0.0011 (13)	0.0045 (13)
C6	0.029 (2)	0.030 (2)	0.0169 (17)	0.0070 (17)	0.0022 (15)	0.0094 (15)
C7	0.028 (2)	0.034 (2)	0.0130 (17)	0.0087 (17)	−0.0044 (14)	0.0041 (15)
C8	0.0205 (18)	0.033 (2)	0.0176 (17)	0.0043 (16)	−0.0045 (14)	−0.0012 (15)
C9	0.0181 (17)	0.0249 (18)	0.0171 (16)	0.0017 (14)	−0.0004 (13)	0.0005 (14)
C10	0.0163 (16)	0.0159 (16)	0.0135 (15)	0.0049 (13)	0.0007 (12)	−0.0016 (12)
C11	0.0220 (18)	0.0237 (19)	0.0182 (17)	0.0009 (15)	−0.0047 (14)	0.0001 (14)
C12	0.055 (3)	0.023 (2)	0.032 (2)	0.0088 (19)	−0.012 (2)	−0.0055 (17)
C13	0.047 (3)	0.047 (3)	0.049 (3)	0.022 (3)	−0.006 (2)	−0.013 (3)

### Geometric parameters (Å, °)

**Table:**

Pb1—O1	2.377 (3)	C4—C5	1.400 (5)
Pb1—O1^i^	2.490 (2)	C4—H4	0.9500
Pb1—O2^ii^	3.145 (3)	C5—C10	1.421 (5)
Pb1—O3	2.382 (3)	C5—C6	1.422 (5)
Pb1—O4	2.761 (3)	C6—C7	1.359 (6)
Pb1—O5	2.696 (3)	C6—H6	0.9500
Pb1—N1	2.643 (3)	C7—C8	1.404 (5)
O1—C1	1.280 (4)	C7—H7	0.9500
O2—C1	1.230 (4)	C8—C9	1.376 (5)
O3—C11	1.276 (4)	C8—H8	0.9500
O4—C11	1.226 (4)	C9—C10	1.415 (5)
O5—C13	1.416 (6)	C9—H9	0.9500
O5—H5	0.84 (4)	C11—C12	1.505 (5)
N1—C2	1.324 (4)	C12—H12A	0.9800
N1—C10	1.372 (4)	C12—H12B	0.9800
C1—C2	1.521 (5)	C12—H12C	0.9800
C2—C3	1.403 (5)	C13—H13A	0.9800
C3—C4	1.375 (5)	C13—H13B	0.9800
C3—H3	0.9500	C13—H13C	0.9800
			
O1—Pb1—O3	77.17 (10)	C4—C3—H3	120.7
O1—Pb1—O1^i^	63.88 (10)	C2—C3—H3	120.7
O3—Pb1—O1^i^	75.27 (9)	C3—C4—C5	119.6 (3)
O1—Pb1—N1	64.02 (8)	C3—C4—H4	120.2
O3—Pb1—N1	95.46 (10)	C5—C4—H4	120.2
O1^i^—Pb1—N1	127.85 (8)	C4—C5—C10	118.4 (3)
O1—Pb1—O5	71.99 (9)	C4—C5—C6	122.9 (3)
O3—Pb1—O5	146.04 (8)	C10—C5—C6	118.6 (3)
O1^i^—Pb1—O5	78.63 (8)	C7—C6—C5	120.8 (4)
N1—Pb1—O5	83.70 (9)	C7—C6—H6	119.6
O1—Pb1—O4	110.27 (9)	C5—C6—H6	119.6
O3—Pb1—O4	49.71 (8)	C6—C7—C8	120.4 (3)
O1^i^—Pb1—O4	122.70 (8)	C6—C7—H7	119.8
N1—Pb1—O4	78.17 (9)	C8—C7—H7	119.8
O5—Pb1—O4	157.89 (8)	C9—C8—C7	120.9 (4)
O1—Pb1—O2^i^	105.53 (7)	C9—C8—H8	119.5
O3—Pb1—O2^i^	76.37 (9)	C7—C8—H8	119.5
O1^i^—Pb1—O2^i^	42.37 (7)	C8—C9—C10	119.7 (3)
N1—Pb1—O2^i^	168.31 (8)	C8—C9—H9	120.2
O5—Pb1—O2^i^	98.35 (8)	C10—C9—H9	120.2
O4—Pb1—O2^i^	101.97 (8)	N1—C10—C9	119.2 (3)
C1—O1—Pb1	126.7 (2)	N1—C10—C5	121.2 (3)
C1—O1—Pb1^i^	115.5 (2)	C9—C10—C5	119.5 (3)
Pb1—O1—Pb1^i^	116.12 (10)	O4—C11—O3	122.0 (4)
C11—O3—Pb1	102.3 (2)	O4—C11—C12	120.4 (3)
C11—O4—Pb1	85.6 (2)	O3—C11—C12	117.6 (3)
C13—O5—Pb1	117.1 (3)	C11—C12—H12A	109.5
C13—O5—H5	110 (4)	C11—C12—H12B	109.5
Pb1—O5—H5	112 (4)	H12A—C12—H12B	109.5
C2—N1—C10	118.3 (3)	C11—C12—H12C	109.5
C2—N1—Pb1	114.6 (2)	H12A—C12—H12C	109.5
C10—N1—Pb1	126.5 (2)	H12B—C12—H12C	109.5
O2—C1—O1	125.0 (3)	O5—C13—H13A	109.5
O2—C1—C2	119.5 (3)	O5—C13—H13B	109.5
O1—C1—C2	115.5 (3)	H13A—C13—H13B	109.5
N1—C2—C3	123.8 (3)	O5—C13—H13C	109.5
N1—C2—C1	116.9 (3)	H13A—C13—H13C	109.5
C3—C2—C1	119.3 (3)	H13B—C13—H13C	109.5
C4—C3—C2	118.6 (3)		
			
O3—Pb1—O1—C1	116.0 (3)	O5—Pb1—N1—C10	−110.9 (3)
O1^i^—Pb1—O1—C1	−164.3 (3)	O4—Pb1—N1—C10	56.4 (3)
N1—Pb1—O1—C1	13.3 (3)	O2^i^—Pb1—N1—C10	148.3 (3)
O5—Pb1—O1—C1	−78.4 (3)	Pb1—O1—C1—O2	169.7 (3)
O4—Pb1—O1—C1	78.3 (3)	Pb1^i^—O1—C1—O2	5.3 (4)
O2^i^—Pb1—O1—C1	−172.3 (3)	Pb1—O1—C1—C2	−12.1 (4)
O3—Pb1—O1—Pb1^i^	−79.70 (12)	Pb1^i^—O1—C1—C2	−176.6 (2)
O1^i^—Pb1—O1—Pb1^i^	0.0	C10—N1—C2—C3	2.0 (5)
N1—Pb1—O1—Pb1^i^	177.62 (15)	Pb1—N1—C2—C3	−170.2 (3)
O5—Pb1—O1—Pb1^i^	85.90 (12)	C10—N1—C2—C1	−175.7 (3)
O4—Pb1—O1—Pb1^i^	−117.41 (11)	Pb1—N1—C2—C1	12.1 (4)
O2^i^—Pb1—O1—Pb1^i^	−8.01 (13)	O2—C1—C2—N1	176.4 (3)
O1—Pb1—O3—C11	−134.9 (3)	O1—C1—C2—N1	−1.8 (5)
O1^i^—Pb1—O3—C11	159.2 (3)	O2—C1—C2—C3	−1.3 (5)
N1—Pb1—O3—C11	−73.1 (3)	O1—C1—C2—C3	−179.6 (3)
O5—Pb1—O3—C11	−159.9 (2)	N1—C2—C3—C4	−3.5 (6)
O4—Pb1—O3—C11	−3.6 (2)	C1—C2—C3—C4	174.1 (3)
O2^i^—Pb1—O3—C11	115.4 (3)	C2—C3—C4—C5	1.7 (6)
O1—Pb1—O4—C11	55.1 (2)	C3—C4—C5—C10	1.4 (6)
O3—Pb1—O4—C11	3.7 (2)	C3—C4—C5—C6	−179.8 (4)
O1^i^—Pb1—O4—C11	−16.2 (3)	C4—C5—C6—C7	−178.2 (4)
N1—Pb1—O4—C11	111.5 (2)	C10—C5—C6—C7	0.6 (6)
O5—Pb1—O4—C11	147.1 (3)	C5—C6—C7—C8	−0.8 (6)
O2^i^—Pb1—O4—C11	−56.6 (2)	C6—C7—C8—C9	1.0 (6)
O1—Pb1—O5—C13	−157.6 (3)	C7—C8—C9—C10	−1.0 (6)
O3—Pb1—O5—C13	−131.9 (3)	C2—N1—C10—C9	−180.0 (3)
O1^i^—Pb1—O5—C13	−91.6 (3)	Pb1—N1—C10—C9	−8.8 (4)
N1—Pb1—O5—C13	137.7 (3)	C2—N1—C10—C5	1.4 (5)
O4—Pb1—O5—C13	102.7 (4)	Pb1—N1—C10—C5	172.5 (2)
O2^i^—Pb1—O5—C13	−53.9 (3)	C8—C9—C10—N1	−177.9 (3)
O1—Pb1—N1—C2	−12.5 (2)	C8—C9—C10—C5	0.8 (5)
O3—Pb1—N1—C2	−85.3 (2)	C4—C5—C10—N1	−3.0 (5)
O1^i^—Pb1—N1—C2	−9.8 (3)	C6—C5—C10—N1	178.1 (3)
O5—Pb1—N1—C2	60.5 (2)	C4—C5—C10—C9	178.3 (3)
O4—Pb1—N1—C2	−132.2 (2)	C6—C5—C10—C9	−0.6 (5)
O2^i^—Pb1—N1—C2	−40.3 (5)	Pb1—O4—C11—O3	−6.2 (4)
O1—Pb1—N1—C10	176.1 (3)	Pb1—O4—C11—C12	176.4 (4)
O3—Pb1—N1—C10	103.2 (3)	Pb1—O3—C11—O4	7.4 (5)
O1^i^—Pb1—N1—C10	178.8 (2)	Pb1—O3—C11—C12	−175.2 (3)

Symmetry codes: (i) −*x*+1, −*y*+1, −*z*+1; (ii) *x*−1, *y*, *z*.

### Hydrogen-bond geometry (Å, °)

**Table:**

*D*—H···*A*	*D*—H	H···*A*	*D*···*A*	*D*—H···*A*
O5—H5···O3^i^	0.84 (4)	1.89 (4)	2.685 (4)	158 (5)

Symmetry codes: (i) −*x*+1, −*y*+1, −*z*+1.

## References

[bb1] Barbour, L. J. (2001). *J. Supramol. Chem.* **1**, 189–191.

[bb2] Bruker (2009). *APEX2* and *SAINT* Bruker AXS Inc., Madison, Wisconsin, USA.

[bb3] Mohammadnezhad, G., Ghanbarpour, A. R., Amini, M. M. & Ng, S. W. (2010). *Acta Cryst.* E**66**, m946. 10.1107/S1600536810027509 PMC3007439 21588176

[bb4] Sheldrick, G. M. (1996). *SADABS* University of Göttingen, Germany.

[bb5] Sheldrick, G. M. (2008). *Acta Cryst.* A**64**, 112–122. 10.1107/S0108767307043930 18156677

[bb6] Westrip, S. P. (2010). *J. Appl. Cryst.* **43**, 920–925.

